# The exit interview as a proxy measure of malaria case management practice: sensitivity and specificity relative to direct observation

**DOI:** 10.1186/s12913-014-0628-8

**Published:** 2014-12-03

**Authors:** Justin Pulford, Peter M Siba, Ivo Mueller, Manuel W Hetzel

**Affiliations:** Papua New Guinea Institute of Medical Research (PNGIMR), PO Box 60, Goroka, EHP 441 Papua New Guinea; School of Population Health, The University of Queensland, Herston, Qld 4006 Australia; Barcelona Centre for International Health Research (CRESIB, Hospital Clínic-Universitat de Barcelona), Barcelona, Spain; Walter and Eliza Hall Institute of Medical Research, Melbourne, Australia; Swiss Tropical and Public Health Institute, PO Box, 4002 Basel, Switzerland; University of Basel, Petersplatz 1, 4003 Basel, Switzerland

**Keywords:** Malaria, Exit interview, Direct observation, Patient recall, Adherence

## Abstract

**Background:**

This paper aims to assess the sensitivity and specificity of exit interviews as a measure of malaria case management practice as compared to direct observation.

**Methods:**

The malaria case management of 1654 febrile patients attending 110 health facilities from across Papua New Guinea was directly observed by a trained research officer as part of a repeat cross sectional survey. Patient recall of 5 forms of clinical advice and 5 forms of clinical action were then assessed at service exit and statistical analyses on matched observation/exit interview data conducted.

**Results:**

The sensitivity of exit interviews with respect to clinical advice ranged from 36.2% to 96.4% and specificity from 53.5% to 98.6%. With respect to clinical actions, sensitivity of the exit interviews ranged from 83.9% to 98.3% and specificity from 70.6% to 98.1%.

**Conclusion:**

The exit interview appears to be a valid measure of objective malaria case management practices such as the completion of a diagnostic test or the provision of antimalarial medication, but may be a less valid measure of low frequency, subjective practices such as the provision of malaria prevention advice.

## Background

Many countries have introduced revised malaria case management protocols over the past decade in light of increasing drug resistance to older anti-malarials and the emergence of affordable malaria rapid diagnostic tests (RDTs). Protocol revisions typically reflect current World Health Organisation guidelines recommending all fever or suspected malaria cases be tested for malaria infection by microscopy or RDT, with some form of artemisinin-combination therapy provided to all confirmed malaria cases [1]. Monitoring health worker practice is an important means by which a national or regional program can determine the success (or otherwise) of implementing a revised malaria case management protocol. High rates of health worker adherence are required if the benefits of the new protocol are to be realised and the consequences of poor adherence, such as misdiagnosis, over- or under-treatment and the accelerated development of drug resistance, averted. As health worker practice is frequently inconsistent with malaria case management protocols [2-5], interventions designed to improve health worker adherence are often necessary e.g. [6-8]. Assessing the impact of any such intervention further requires a means by which to monitor health worker practice.

While monitoring malaria case management practice is necessary in relevant program evaluation or intervention studies, there has been little consideration given to methods of assessment in the published literature. A number of methods have been used, including direct observation of clinical case management [9,10], exit interviews with febrile patients conducted immediately after service discharge [6,11], reviews of medical records [12] and health worker interviews [13]. However, the authors are aware of no studies that have sought to examine the relative strengths and weaknesses of each assessment method or examined their relative validity in the context of malaria case management. Direct methods such as audio- or video-recording, observation by a trained observer or the use of a ‘standardised’ patient are considered gold standard measures of health worker practice [14], yet they may also be time consuming, labour intensive, intrusive and can promote social desirability bias (e.g. health workers modifying their usual practice when being observed or recorded). Accordingly, proxy measures of health worker practice, such as patient self-report (at service exit or sometime thereafter), health worker self-report or medical file review are often preferred. Proxy measures are less intrusive, less costly and may reduce social desirability bias, although the degree to which they accurately reflect health worker practice remains open to question. Several studies have reported divergent findings when comparing one form of proxy measurement with another [15-17] or when comparing a direct measurement with a proxy [18,19]. A review paper designed to identify valid proxy measures of clinical behaviour concluded patient self-report demonstrated greater accuracy than either medical file review or clinician self-report when compared to a direct measure [14]. However, the authors further concluded the evidence base for all three forms of proxy measure is very limited, due in large part to the use of inappropriate statistical methods, and that direct measures themselves may not be uniformly valid as a gold standard across all aspects of health worker practice.

Given the lack of investigation into valid measures of malaria case management practice specifically, and the dearth of statistically sound comparisons of direct versus proxy measures of clinical practice generally, this paper aims to compare a direct and a proxy measure of health worker practice in the context of malaria case management. Reported analyses draw on matched data obtained by direct observation of malaria case management and structured interviews (exit interview) conducted with the same patients or their caregivers immediately following service discharge from health facilities in Papua New Guinea (PNG). The analysis plan followed that recommended by Dickinson et al. [20] for comparing direct and proxy measures of clinical behavior (item by item comparisons), including a measure of sensitivity to answer the question: what proportion of actions that were actually performed and recorded by direct observation were identified by the proxy (exit interview)? And a measure of specificity to answer the question: what proportion of actions that were *not* performed or recorded by direct observation were equally *not* recorded as performed by the proxy? Factors potentially predictive of accurate patient/caregiver recall are also examined via logistic regression.

## Method

Data were collected during a repeat cross sectional survey of randomly selected health facilities from across PNG. This paper presents combined data from the first three of these surveys completed in the years’ 2010, 2011 and 2012 (the only surveys completed at the time of analysis). All three surveys were conducted as part of a five year evaluation of the PNG National Malaria Control Program. A full description of the evaluation program, including a detailed description of the health facility survey methodology, is presented elsewhere [21]. The following description is a summarized version of this previously published account.

### Study sample

The study sample for each cross sectional survey consisted of two health centres and up to four aid posts selected from each of 20 PNG provinces, using a simple random sampling procedure repeated for each survey. The sampling frame in each was a list of all operational public-sector health centres nationwide as provided by the National Department of Health (N = 689). Aid posts were randomly selected on site at participating health centres. In this paper, study samples from the 2010, 2011 and 2012 surveys have been combined into a single dataset for analysis. Cases were included in the final dataset if matched direct observation and exit interview data were available. Health centres and aid posts are the main providers of primary care in PNG. The median number of health workers employed at health centres in PNG is eight and the median number at aid posts is one, almost all of whom are either nurses or community health workers [22].

### Procedure

Each survey was carried out from June to November and was conducted by trained survey teams working simultaneously at different sites. The training programme for survey staff spanned 10 days and included intensive instruction and practice on the methods of direct observation and exit interview. Members of each survey team spent between three to five days at each participating health centre and up to one day at each participating aid post. Oral informed consent was sought from the officer in charge at all participating health facilities and from all participating clinicians and patients prior to observation/interview. Patients were considered eligible for participation if they were presenting with febrile symptoms or reported a recent history of fever. Eligible patients were identified upon first contact with a health worker or, if circumstances allowed, by screening in the waiting area prior to first contact with a health worker. Consenting participants were observed/interviewed consecutively and were retained in the study sample irrespective of whether they were subsequently diagnosed with malaria or not. The study was approved and granted ethical clearance by the Medical Research Advisory Committee of PNG (MRAC No. 10.12; 26 Feb 2010).

### Measures

The method of direct observation was undertaken by a trained research officer who passively observed the management of fever patients from the point of initial contact with a health professional until service exit or admission onto a treatment ward. During the course of this observation, the research officer recorded whether specified actions did or did not occur as well as the content of specific actions (e.g. whether an antimalarial was prescribed and, if yes, what type of antimalarial) on a structured checklist. The checklist was divided into discrete sections including diagnosis, prescription and treatment counselling and was informed by input from experienced medical- and medical research- professionals. The exit interview took the form of a structured questionnaire administered by a trained research officer to fever patients at the time of service discharge. The questionnaire included a range of open and closed questions pertaining to his or her recall of clinical information/clinical practice.

In this paper, the analysis is based on patient recall (as measured at exit interview) of ten clinical variables: five pertaining to clinical advice that the current PNG national malaria treatment protocol recommends all health workers provide to malaria patients [23] and five pertaining to clinical actions routinely associated with malaria case management. The five ‘advice’ variables include: 1) ‘dosage regimen’ , advice on when, how many and over how many days, prescribed antimalarial tablets should be consumed; 2) ‘dietary’ , advice to consume antimalarial tablets with food or with certain types of food; 3) ‘adverse effects’ , advice on potential side effects of prescribed antimalarial drugs; 4) ‘re-engagement’, advice on when, and under what circumstances, a patient should return to the health facility (e.g. if symptoms persist or worsen); and 5) ‘malaria prevention’ , advice on how to protect oneself and/or one’s family against malaria infection (e.g. sleep under a mosquito net every night). The five ‘action’ variables included: 1) ‘diagnosis’ , whether the health worker completed a malaria rapid diagnostic test or took a malaria blood slide; 2) ‘antimalarial prescription’ , whether the health worker prescribed an antimalarial drug; 3) ‘sulphadoxine-pyrimethamine (SP) prescription’ , whether the health worker prescribed SP (included as a proxy measure of patients’ ability to recall a specific drug as opposed to a class of drug. SP was selected as it was the most frequently prescribed antimalarial in the study sample); 4) ‘First dose ingested’ , whether the patient consumed the first dose of the prescribed antimalarial(s) at the health centre; and 5) ‘take away dose’ , whether the patient was provided with antimalarial medication to take home.

### Data analysis

All data were double entered into DMSys version 5.1 (Sigma Soft International). Stata/SE version 12 was used for statistical analysis, including tests of sensitivity and specificity with 95% confidence intervals (CIs) calculated for all summary statistics. Sensitivity was defined as the number of cases in which the patient/caregiver correctly reported that a specified advice/action was provided divided by the total number of cases in which the specified advice/action was observed to have occurred. Specificity was defined as the number of exit interviews in which the patient/caregiver correctly reported that the specified advice/action was not provided divided by the total number of cases in which the specified advice/action was observed not to have occurred. The calculation of all CIs was adjusted for possible clustering at the health facility level by using the Stata ‘svy’ command in which health facilities were defined as the primary sampling unit. Factors potentially predictive of accurate recall (of both clinical-advice and -action) were examined by logistic regression. The outcome variable was whether a patient/caregiver accurately recalled whether the specified action/advice took place (yes/no). In each analysis, the sample was limited to the number of patients for whom the specified advice/action was observed to have occurred. Patient/caregiver recall was scored accurate (yes) if he/she stated the observed advice/action took place. Predictor variables included patient age (<5 years/5 + years), patient sex, interviewee status (patient/caregiver) and the consultation duration (<17 minutes/17+ minutes; 17 minutes being the mean consultation duration).

## Results

### Study sample

Across the three surveys, the clinical case management of 1,932 febrile patients was directly observed by a trained observer and exit interviews conducted with 1,796 febrile patients. Of these, matched observation/exit interview data were available for up to 1654 febrile case management patients (the total number of matched pairs available varied by variable type). These 1,654 patients collectively attended 110 health facilities, 90.9% (100/110) of which were health centres. Sex and age characteristics of the sample by survey year and overall, along with the geographical region in which treatment was sought, are presented in Table 1.

**Table 1:** **Selected characteristics of the study sample by survey year and overall**

**Characteristic**		**2010**		**2011**		**2012**		**Overall**	
		**n**	**%**	**n**	**%**	**n**	**%**	**n**	**%**
Sex	Male	286	51.8	337	50.8	233	53.8	856	51.9
	Female	266	48.2	327	49.2	200	46.2	793	48.1
Age	0 - 4 yrs	249	45.0	343	51.3	210	48.5	802	48.5
	5 – 14 yrs	129	23.3	124	18.6	96	22.2	349	21.1
	15+ yrs	175	31.7	201	30.1	127	29.3	503	30.4
Region	Southern	152	27.5	151	22.6	151	34.9	454	27.5
	Highlands	141	25.5	136	20.4	83	19.2	360	21.8
	Momase	146	26.4	198	29.6	127	29.3	471	28.5
	Islands	114	20.6	183	27.4	72	16.6	369	22.3

### Sensitivity and specificity of exit interview measures

Table 2 presents the sensitivity and specificity of exit interviews relative to direct observation on specified forms of clinical advice and clinical actions. As shown, the sensitivity of exit interviews with respect to clinical advice ranged from 36.2% (advice pertaining to potential adverse effects of a prescribed medication) to 96.4% (advice pertaining to the dosage regimen of the prescribed medication) and specificity from 53.5% (dosage regimen) to 98.6% (adverse effects). With respect to clinical actions, sensitivity of the exit interviews ranged from 83.9% (prescription of SP) to 98.3% (whether antimalarial medication was given to the patient to take home) and specificity from 70.6% (prescription of SP) to 98.1% (whether an RDT or bloodslide completed).

**Table 2:** **Sensitivity and specificity of exit interviews compared to direct observation**

**Advice/action**	**Observations** ^**1**^	**Occurrences** ^**2**^	**Sensitivity**	**Specificity**
	**N**	**N**	**% (95% CI)**	**% (95% CI)**
Advice:				
Dosage regimen	1229	1027	96.4 (93.6, 98.0)	53.5 (40.2, 66.2)
Dietary	1251	81	54.4 (41.9, 66.2)	94.1 (92.0, 95.6)
Adverse effects	1253	36	36.2 (16.8, 61.2)	98.6 (97.6, 99.2)
Re-engagement	1643	318	57.9 (49.4, 65.9)	75.2 (70.3, 79.5)
Malaria prevention	1644	185	69.2 (60.5, 76.7)	96.9 (95.6, 97.7)
Action:				
RDT/BS completed^3^	1556	445	94.0 (88.8, 96.8)	98.1 (95.5, 99.1)
Prescription made	1645	1409	91.7 (87.8, 94.4)	97.9 (92.7, 99.4)
SP prescribed^4^	1266	909	83.9 (79.0, 87.7)	70.6 (63.4, 83.3)
1st dose ingested	1068	548	95.5 (93.0, 97.1)	95.0 (92.4, 96.8)
Take away dose	1067	737	98.3 (97.1, 98.9)	88.2 (81.7, 92.6)

^1^Observations = the number of participants included in the analysis (i.e. the number of observations carried out); ^2^Occurrences = the number of times the specified advice/action was observed to have occurred (e.g. in 1027/1229 observed clinical case management cases the participant was provided dosage regimen advice); ^3^RDT = rapid diagnostic test, BS = bloodslide; ^4^SP = sulphadoxine-pyrimethamine, SP was the most frequently prescribed antimalarial across the three survey samples and is used here as a measure of how accurately patients/caregivers can recall specific antimalarial medications.

### Factors associated with accurate patient recall

Results of the logistic regression are presented in Tables 3 and 4. Few statistically significant associations between the respective outcome and predictor variables were found. Exceptions included statistically significant associations between: interviewee status and accurate recall of dietary- and adverse effect advice and use of RDT or bloodslide; patient sex and accurate recall of adverse effects advice and antimalarial prescription; and between consultation time and accurate recall of adverse effects advice.

**Table 3:** **Factors associated with accurate recall of treatment advice**

**Factor**	**Dosage regimen**		**Dietary advice**		**Adverse effects**		**Re-engagement**		**Malaria prevention**	
	**OR (95% CI)**	***p***	**OR (95% CI)**	***p***	**OR (95% CI)**	***p***	**OR (95% CI)**	***p***	**OR (95% CI)**	***p***
Interviewee										
Patient	1.00		1.00		1.00		1.00		1.00	
Caregiver	1.1 (0.4, 3.0)	0.78	0.3 (0.1, 0.9)	0.03	0.1 (<0.1, 1.0)	0.05	0.9 (0.4, 1.9)	0.82	2.4 (0.9, 6.4)	0.07
Patient sex										
Male	1.00		1.00		1.00		1.00		1.00	
Female	0.7 (0.4, 1.2)	0.24	1.5 (0.6, 4.1)	0.39	0.1 (<0.1, 0.9)	0.04	0.8 (0.5, 1.2)	0.26	0.9 (0.5, 1.7)	0.83
Patient age										
< 5 yrs	1.00		1.00		1.00		1.00		1.00	
5 yrs +	1.1 (0.6, 2.0)	0.86	1.0 (0.4, 2.5)	0.99	2.6 (0.3, 19.5)	0.33	1.0 (0.5, 1.8)	0.97	1.8 (0.8, 4.3)	0.17
Consult time										
<17 mins	1.00		1.00		1.00		1.00		1.00	
17 mins +	1.2 (0.6, 2.1)	0.63	0.7 (0.2, 2.1)	0.51	0.1 (<0.1, 0.8)	0.04	1.5 (0.8, 2.8)	0.19	0.6 (0.3, 1.4)	0.26

**Table 4:** **Factors associated with accurate recall of treatment actions**

**Factor**	**Diagnosis**		**AM prescription** ^**1**^		**SP prescription** ^**2**^		**1st dose ingested**		**Take away dose**	
	**OR (95% CI)**	***p***	**OR (95% CI)**	***p***	**OR (95% CI)**	***p***	**OR (95% CI)**	***p***	**OR (95% CI)**	***p***
Interviewee										
Patient	1.00		1.00		1.00		1.00		1.00	
Caregiver	2.9 (1.0, 8.3)	0.05	1.3 (0.8, 2.2)	0.30	1.1 (0.7, 1.8)	0.71	2.0 (0.6, 6.6)	0.23	0.7 (0.1, 3.8)	0.72
Patient sex										
Male	1.00		1.00		1.00		1.00		1.00	
Female	1.5 (0.5, 4.4)	0.43	0.6 (0.5, 0.9)	0.01	1.1 (0.8, 1.7)	0.50	2.6 (1.0, 7.0)	0.06	2.2 (0.7, 7.1)	0.20
Patient age										
< 5 yrs	1.00		1.00		1.00		1.00		1.00	
5 yrs +	1.7 (0.8, 3.9)	0.19	1.7 (1.0, 2.8)	0.06	1.1 (0.6, 1.7)	0.84	1.0 (0.3, 3.6)	0.96	1.3 (0.3, 6.1)	0.77
Consult time										
<17 mins	1.00		1.00		1.00		1.00		1.00	
17 mins +	1.3 (0.6, 3.2)	0.52	1.3 (0.8, 2.0)	0.32	1.0 (0.7, 1.6)	0.83	1.1 (0.4, 2.6)	0.90	0.8 (0.3, 2.4)	0.66

^1^AM = antimalarial; ^2^SP = sulphadoxine-pyrimethamine.

## Discussion

The reported findings indicate that the exit interview was a poor proxy measure of malaria case management practice in regards to the provision of clinical advice. Sensitivity was as low as 36.2% on one ‘advice’ measure (adverse effects) and less than 60% on two others (diet and re-engagement), indicating patients’ frequently failed to recall the provision of clinical advice when such advice had been given. Specificity was higher than sensitivity on four out of five ‘advice’ measures and above 90% on three. While this indicates high concordance between the exit interview and direct observation in terms of detecting the non-provision of clinical advice, this finding is tempered by the fact that the forms of clinical advice measured were rarely provided at all. Thus, the number of ‘false positives’ detected by the exit interview may have been small relative to the number of correctly identified false cases (i.e. non-provision of clinical advice), but may still have exceeded or closely matched the number of correctly identified positive cases (i.e. provision of clinical advice). The ability of the exit interview to reliably detect the provision of clinical advice, therefore, is highly questionable if direct observation is taken as a reliable gold standard bench-mark.

Having said this, the exit interview proved to be a valid proxy measure of malaria case management practice in regards to the provision of clinical actions. Four out of the five ‘action’ measures had a sensitivity or specificity greater than 90% and in three out of the five measures both sensitivity and specificity was greater than 90%. The high frequency with which the specified actions occurred also indicates that there were few cases of false negatives or false positives relative to the number of actions correctly identified by exit interview (i.e. either having occurred or not occurred). Thus, in sum, the reported findings indicate that for frequently occurring, highly objective measures such as the prescription of an antimalarial or the use of a diagnostic test, the exit interview performs well as a proxy measure of malaria case management practice. However, for less frequent, more subjective measures such as the provision of malaria prevention advice or dietary information, the exit interview performs less well. The logistic regression analyses suggest recall accuracy is not consistently influenced by interviewee status, patient age or sex or consultation time. Where statistically significant associations were reported, the respective sample sizes were typically very low undermining confidence in the result.

These findings are somewhat consistent with the extant evidence-base. A number of studies have reported poor to moderate patient recall (as measured by exit interview) of the provision of health behaviour advice when compared to a direct measure of health worker practice [16,18,24]. In one study, patient recall on nine ‘advice’ domains (ranging from diet and exercise to seatbelt use and HIV prevention) exceeded 50% on only three domains and was as low as 11% for recall of substance use advice [18]. In another, and converse to what was reported in this paper, parent recall of diet and physical activity advice provided to their child in the course of a paediatric consultation was found to have relatively high sensitivity (70-96%), but low specificity (43%-78%) [16]. Other studies that have examined proxy measures of more objective clinical practices, such as measuring blood pressure or providing medication, have reported greater agreement with direct measurements [25,26]. Nevertheless, the available literature is not neatly divided along these lines. High levels of agreement between proxy and direct measurements have been reported for the provision of health behaviour advice in some instances [27] and proxy measures of objective clinical practices such as components of a physical exam have not always uniformly produced high sensitivity or specificity [19]. Drawing firm conclusions in regard to the validity of the exit interview as a proxy measure of clinical practice continues to be hampered by the dearth of statistically appropriate validation studies. This paper and others [16] published subsequent to the review paper which first highlighted this limitation [14] are improving the stock of available evidence, but further research is necessary. As the emerging evidence base is beginning to challenge the validity of the exit interview as a reliable measure of low frequency, subjective clinical practices then this should be considered an area of research priority. Until such time, researchers could consider employing multiple proxy measures of clinical practice (e.g. exit interview, file review, health worker interview) and triangulating the resulting data in order to improve confidence in the reported findings as has been previously advocated [28,29].

Future research in this area could further benefit by accounting for health literacy and other aspects of health communication when assessing the utility of proxy measures of clinical practice. A patient’s health literacy, defined as “The degree to which individuals can obtain, process, understand, and communicate about health-related information needed to make informed health decisions” [30] pg. 16, is an individual-level construct influenced by literacy and cognitive abilities [31]. However, irrespective of one’s health literacy, comprehension of health information can be influenced by external factors such as the quality of clinician-patient communication and interventions can improve patient understanding and recall [32]. Accordingly, clinical practices centred on verbal communication, such as advice pertaining to when, and under what circumstances one should seek further treatment, may be influenced by a greater range of factors as compared to recall of objective non-verbal actions such as the provision of a diagnostic test. The relative merit of using an exit interview as a proxy measure of clinical practice may, therefore, be dependent on the health literacy of the patient population and the perceived degree to which the quality of health communication may influence patient recall. In settings such as PNG where the patient population is likely to have relatively low health literacy and the health workforce is likely to have relatively undeveloped health communication skills, an exit interview may be a less accurate proxy measure of verbal, subjective clinical practices as compared to other forms of measurement or to the use of an exit interview in a higher income country (where the health literacy of the patient population and the communication skills of the health workforce are likely to be better developed).

The strengths of this study included the use of a statistical design appropriate to validation of a proxy measure of clinical practice, the use of matched data drawn from a nationally representative sample of health facilities and the inclusion of both advice- and action-centred clinical practices. Nevertheless, the reported study was not without limitation. The sample size for the ‘diet’ and ‘adverse effects’ advice measures was problematic due to the low frequency with which either form of advice was actually provided (n = 81 and 36, respectively). This limitation was especially problematic for the logistic regression analysis, the results of which should be considered highly tentative. All confidence intervals (CIs) were also adjusted for possible clustering at the health facility level rather than the health worker level, which would have been preferable. Unfortunately, it was not possible to adjust CIs at the health worker level in this study. Only a minority of health workers employed at any one health centre would have provided clinical treatment to febrile patients during the course of study participation. Finally, this paper only examined one form of proxy measurement (exit interview) and utilised only one form of direct measurement (observation). The reported findings would be usefully complemented by validation studies of other proxy measures (e.g. file review or health worker interview) against direct observation or the same proxy measure against an alternative form of direct measurement (e.g. video- or audio-taping).

## Conclusion

The exit interview appears to be a valid measure of objective malaria case management practices such as the completion of a diagnostic test or the provision of antimalarial medication, but may be a less valid measure of low frequency, subjective practices such as the provision of malaria prevention advice. Further research is needed to establish the validity of the exit interview as a measure of health behaviour advice.
